# Implementation of a Self-Triage Web Application for Suspected COVID-19 and Its Impact on Emergency Call Centers: Observational Study

**DOI:** 10.2196/22924

**Published:** 2020-11-23

**Authors:** Simon Galmiche, Eve Rahbe, Arnaud Fontanet, Aurélien Dinh, François Bénézit, François-Xavier Lescure, Fabrice Denis

**Affiliations:** 1 Emerging Diseases Epidemiology Unit Institut Pasteur Paris France; 2 Unité Pasteur-CNAM Risques Infectieux et Emergents (PACRI) Conservatoire National des Arts et Métiers Paris France; 3 Service de Maladies Infectieuses et Tropicales Hôpital Raymond Poincaré Assistance Publique - Hôpitaux de Paris Garches France; 4 Service de Maladies Infectieuses et Réanimation Médicale Centre Hospitalier Régional et Universitaire Pontchaillou Rennes France; 5 Infectious and Tropical Diseases Department Bichat-Claude Bernard University Hospital and University of Paris Assistance Publique - Hôpitaux de Paris Paris France; 6 Unité Institut National de la Santé et de la Recherche Médicale Paris France; 7 Institut Inter-régional de Cancérologie Jean Bernard Le Mans France

**Keywords:** COVID-19, emergency medical services, emergency call center, questionnaires, application, website, self-triage, digital health, smartphone, mobile phone

## Abstract

**Background:**

We developed a self-triage web application for COVID-19 symptoms, which was launched in France in March 2020, when French health authorities recommended all patients with suspected COVID-19 call an emergency phone number.

**Objective:**

Our objective was to determine if a self-triage tool could reduce the burden on emergency call centers and help predict increasing burden on hospitals.

**Methods:**

Users were asked questions about their underlying conditions, sociodemographic status, postal code, and main COVID-19 symptoms. Participants were advised to call an emergency call center if they reported dyspnea or complete loss of appetite for over 24 hours. Data on COVID-19–related calls were collected from 6 emergency call centers and data on COVID-19 hospitalizations were collected from Santé Publique France and the French Ministry of Health. We examined the change in the number of emergency calls before and after the launch of the web application.

**Results:**

From March 17 to April 2, 2020, 735,419 questionnaires were registered in the study area. Of these, 121,370 (16.5%) led to a recommendation to call an emergency center. The peak number of overall questionnaires and of questionnaires leading to a recommendation to call an emergency center were observed on March 22, 2020. In the 17 days preceding the launch of the web application, emergency call centers in the study area registered 66,925 COVID-19–related calls and local hospitals admitted 639 patients for COVID-19; the ratio of emergency calls to hospitalizations for COVID-19 was 104.7 to 1. In the 17 days following the launch of the web application, there were 82,347 emergency calls and 6009 new hospitalizations for COVID-19, a ratio of 13.7 calls to 1 hospitalization (chi-square test: *P*<.001).

**Conclusions:**

The self-triage web application launch was followed by a nearly 10-fold increase in COVID-19–related hospitalizations with only a 23% increase in emergency calls. The peak of questionnaire completions preceded the peak of COVID-19–related hospitalizations by 5 days. Although the design of this study does not allow us to conclude that the self-triage tool alone contributed to the alleviation of calls to the emergency call centers, it does suggest that it played a role, and may be used for predicting increasing burden on hospitals.

**Trial Registration:**

ClinicalTrials.gov NCT04331171; https://clinicaltrials.gov/ct2/show/NCT04331171

## Introduction

Since February 2020, France has been hit by a severe COVID-19 epidemic that partly overwhelmed health system capacities. At the beginning of the epidemic, the French Ministry of Health recommended all patients with suspected COVID-19 call an emergency call center (Centre 15). As a result, patients experienced long delays before reaching an operator; some of these patients had a condition requiring emergency care. In this context, triage tools preselecting patients who should call the emergency call center may be particularly helpful. Web-based self-triage of symptoms is a growing field and has been shown to improve survival in oncology [1,2]. Past data have shown the feasibility of self-triage by parents of children with influenza-like illnesses, although specificity was weak [3]. Self-triage symptom checkers have higher levels of appropriate triage when used for emergency care, according to a study on symptom checkers that are available in Australia [4]. The use of web-based tools for COVID-19 management is currently increasing [5], but little data are available on self-triage and its impact on health care use. We sought to develop a web-based self-triage tool to optimize triage of patients with COVID-19 in France. A web application [6] was developed and launched while the COVID-19 epidemic was growing in France in March 2020. Our objective was to determine if a self-triage tool for COVID-19 could reduce the burden on emergency call centers and help predict increasing burden on hospitals.

## Methods

The web application was launched on March 17, 2020, via a national media campaign in France including social media, radio, and magazine media. At that time, the French Ministry of Health recommended that all patients with suspected COVID-19 call an emergency call center. The recruitment process via the web application has already been detailed in a previous work [7]. Participants were asked about their postal code, pre-existing conditions, and potential COVID-19 symptoms (fever defined as body temperature >37.7 °C, unusual cough, shortness of breath, sore throat, muscle aches, diarrhea, loss of appetite, fatigue, anosmia, and ageusia). Depending on reported symptoms and underlying conditions, the user was recommended either to stay home and reuse the application in case of evolving symptoms (self-monitoring), or to contact a general practitioner (GP), or to call an emergency call center (if they reported shortness of breath or complete loss of appetite for over 24 hours) [8]. The web application did not offer monitoring of participant adherence to the self-triage recommendation. Access to the web application did not require a login or account creation. The web application did not identify participants who responded several times and did not follow up on participants. Questionnaires were excluded from the analysis if they did not include a postal code or if the completion duration was considered inconsistent (<30 seconds). This study was approved by the French National Health-Data Institute, which reviews ethical conduct of human subject research, data confidentiality, and safety.

We collected data on COVID-19–related calls from 6 emergency call centers that cover some of the most severely COVID-19–affected areas in France (Bas-Rhin, Paris, Hauts-de-Seine, Seine-Saint-Denis, Val-de-Marne), where burden was expected to be highest on emergency call centers, and one area where the web application was advertised through local papers a few days before the nationwide campaign, allowing for an earlier evaluation of impact (Sarthe). Data included calls made before the web application launch, from the day the first COVID-19–related hospitalization following an emergency room (ER) consultation was reported in the study area. That period, starting February 29, 2020, covers the 17 days preceding the launch of the web application. All of the territories covered in the study area had reported their first hospitalization following ER consultation for COVID-19 by March 3, 2020. We collected the same data the 17 days following the launch of the web application. Data regarding daily hospitalizations for COVID-19 following evaluation at an ER in the study area were provided by Santé Publique France and the French Ministry of Health. We compared the ratio of daily emergency center calls reported by emergency call centers to daily hospitalizations for COVID-19 before and after the launch of the web application using a chi-square test.

## Results

**Figure 1:**
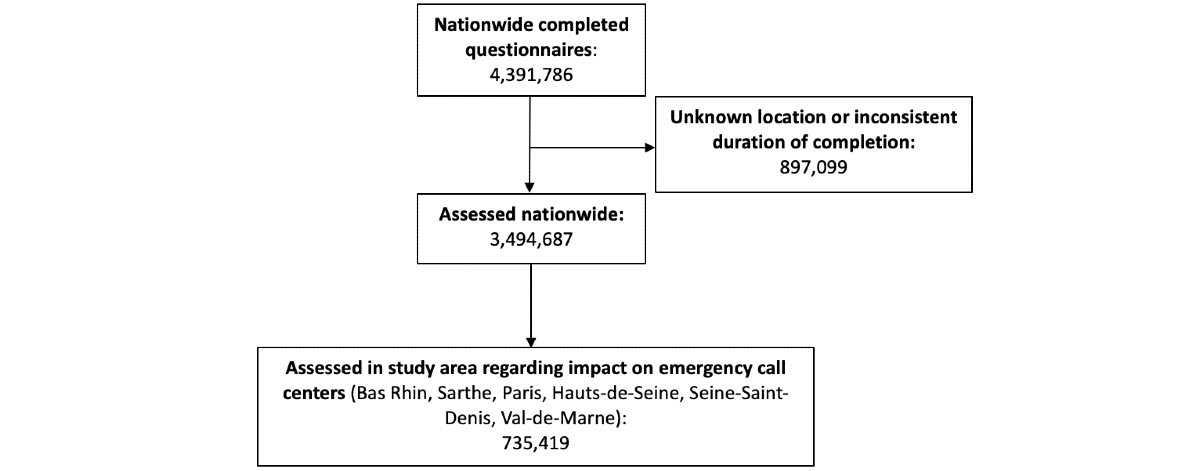
Flowchart of self-triage web application respondents.

**Figure 2:**
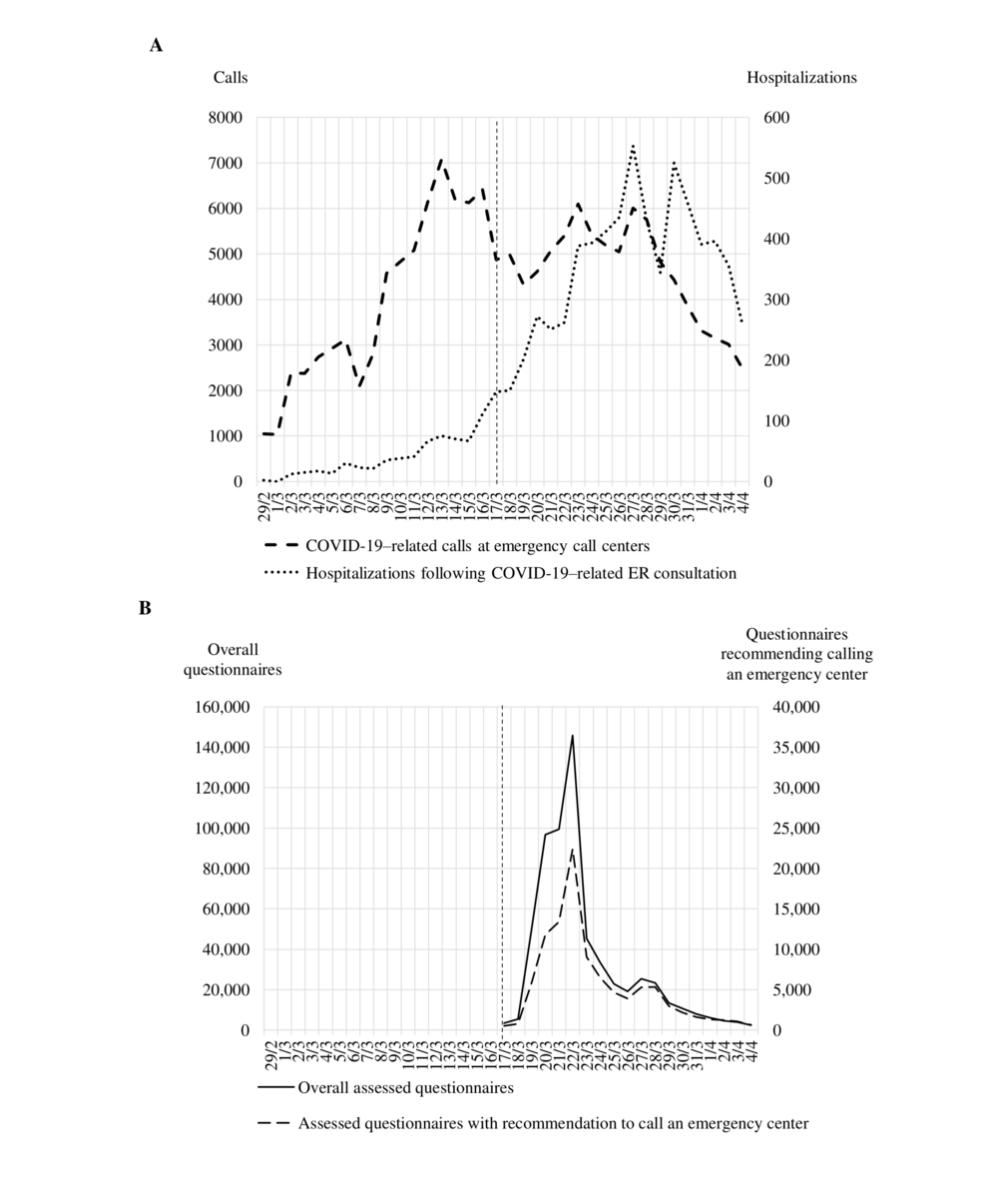
Data from the study area (raw numbers). (A) COVID-19–related calls to emergency call centers and hospitalizations following a COVID-19–related emergency room consultation. (B) Overall assessed questionnaires and the number of recommendations to call an emergency center. The web application was launched March 17, 2020 (dashed line).

From March 17 to April 2, 2020, there were 4,391,786 questionnaires filled out nationwide (Figure 1). Of these, 897,099 questionnaires were excluded from analysis for not including a postal code or inconsistent completion duration. The number of assessed questionnaires represent the number of assessments and not individuals. Among the 3,494,687 assessed questionnaires, 558,236 (16.0%) led to a recommendation to call an emergency call center. In the study area, 735,419 questionnaires were assessed, among which 121,370 (16.5%) led to a recommendation to call an emergency call center. Both the peak of overall questionnaires and that of questionnaires leading to a recommendation to call an emergency center were observed on March 22, 2020 (155,415 and 23,952, respectively; Figure 2).

The first hospitalization for COVID-19 following an ER consultation in the study area was reported on February 29, 2020. The peak of hospitalizations was observed on March 27, with 553 hospitalizations (Figure 2). In the 17 days preceding the launch of the web application, emergency call centers in the study area registered 66,925 COVID-19–related calls and local hospitals admitted 639 patients for COVID-19, a ratio of 104.7 calls to 1 hospitalization. In the 17 days following the launch of the application, there were 82,347 COVID-19–related emergency calls (a 23% increase from the previous period) and 6009 new hospitalizations for COVID-19 (a 9.4-fold increase from the previous period), resulting in a ratio of 13.7 calls to 1 hospitalization (chi-square test: *P*<.001; Figure 2).

## Discussion

The launch of the self-triage web application was followed by a nearly 10-fold increase in COVID-19–related hospitalizations with only a 23% increase in emergency calls, even though the number of completed questionnaires quickly surged, including questionnaires leading to a recommendation to call an emergency center, indicating appropriate use of the tool. Both the peak of overall questionnaires and that of questionnaires leading to a recommendation to call an emergency center happened 5 days after lockdown started in France on March 17, 2020. This is compatible with a maximum incidence rate of SARS-CoV-2 infections one day before lockdown, considering a mean 5-day incubation period for COVID-19 [9,10]. There was a further delay of 5 days between the peak of questionnaire completions and the peak of COVID-19–related hospitalizations, consistent with a mean duration between infection and hospitalization for severe forms of the disease of 10 days, as previously described [11]. It is unknown how many questionnaires were filled out by people with COVID-19. However, the nationwide daily incidence immediately before the lockdown onset on March 17, 2020, was estimated to be between 180,000 and 490,000 in a study by Salje et al [12]. The positive predictive value of general symptoms (eg, dyspnea or loss of appetite) increases in such a high-incidence setting, suggesting a significant share of people reporting symptoms during the surge peaking on March 22 had COVID-19, although more precise evaluation is impossible. It indicates that the self-triage tool could help predict a rise in severe cases and burden on hospitals. This hypothesis needs confirmation should a new surge in COVID-19 cases and related hospitalizations occur.

There are few data regarding the impact of self-triage tools on health care use. A recent systematic review of self-triage symptom checkers for urgent health problems suggested they led to less frequent health care use [13]. Verzantvoort et al [14] reported 67% of patients receiving self-care advice intended to follow the advice. For COVID-19, Judson et al [15] described a dedicated self-triage tool that recommended self-care to 40% of symptomatic patients; that advice was mostly followed, as only 8% of them had an in-person visit in the following 48 hours, suggesting an effective reduction in unnecessary GP or ER visits [15].

The design of the present study does not allow us to conclude that the self-triage tool alone contributed to the alleviation of calls to the emergency call centers. Other interventions, such as the creation of an information hotline for nonurgent COVID-19–related questions, happened soon after the launch of the web application and may have contributed to relieving the burden on emergency call centers. The sudden drop in completed questionnaires following the peak may be related to the drop in infections after lockdown, but may also indicate the influence of media campaigns that promoted the use of the web application. Interpretation of peaks in website usage should therefore be cautious and take into account those campaigns. Altogether, we think that a self-triage tool can be useful in periods of high incidence of COVID-19, when health care use quickly surges and health care providers such as emergency call centers endure a rapidly increasing burden. Helping predict increasing burden on hospitals may also help policy makers and health care providers by informing their response.

## Abbreviations

ER: emergency room
GP: general practitioner
